# Safety and outcomes analysis: transcatheter implantation of autologous angiogenic cell precursors for the treatment of cardiomyopathy

**DOI:** 10.1186/s13287-023-03539-6

**Published:** 2023-10-26

**Authors:** Jane R. Schubart, Amirhossein Zare, Roberto M. Fernandez-de-Castro, Hector Rosario Figueroa, Ina Sarel, Kelly Tuchman, Kaitlyn Esposito, Fraser C. Henderson, Ernst von Schwarz

**Affiliations:** 1 Penn State College of Medicine, Pennsylvania State University, Hershey, PA USA; 2 Northern Ontario School of Medicine, Ontario, CA USA; 3 Invasive Cardiovascular Laboratory, Dominican Cardiovascular Center, Santo Domingo, Dominican Republic; 4 Clinica Union Medica, Santiago, Dominican Republic; 5 The Metropolitan Neurosurgery Group, LLC, 1010 Wayne Ave Suite 420, Silver Spring, MD 20910 USA; 6 The Bobby Jones Chiari Syringomyelia Foundation, New York, NY USA; 7 Department Neurosurgery, University of Maryland School of Medicine, Baltimore, MD USA; 8 Hemostemix Inc, Calgary, CA Canada; 9 School of Medicine, University of California Los Angeles (UCLA), Los Angeles, CA USA; 10 Cedars Sinai Medical Center, Los Angeles, CA USA

**Keywords:** Autologous hematopoietic stem cell, VEGF, Angiogenin, Cytokine IL-8, NF-kB, Anti-apoptosis, Cardiomyopathy, Angiogenic stem cell, Angiogenesis

## Abstract

**Background:**

Stem cell transplantation is an emerging therapy for severe cardiomyopathy, proffering stem cell recruitment, anti-apoptosis, and proangiogenic capabilities. Angiogenic cell precursors (ACP-01) are autologous, lineage-specific, cells derived from a multipotent progenitor cell population, with strong potential to effectively engraft, form blood vessels, and support tissue survival and regeneration.

**Methods:**

This IRB approved outcome analysis reports upon 74 consecutive patients who failed medical management for severe cardiomyopathy, and were selected to undergo transcatheter intramyocardial or intracoronary implantation of ACP-01. Serious adverse events (SAEs) were reported. Cell analysis was conducted for each treatment. The left ventricular ejection fraction (LVEF) was measured by multi-gated acquisition scan (MUGA) or echocardiogram at 4 months ± 1.9 months and 12 months ± 5.5 months. Patients reported quality of life statements at 6 months (± 5.6 months).

**Results:**

Fifty-four of 74 patients met requirements for inclusion (48 males and five females; age 68.1 ± 11.3 years). The mean treatment cell number of 57 × 10^6^ ACP-01 included 7.7 × 10^6^ CD34 + and 21 × 10^6^ CD31 + cells with 97.6% viability. SAEs included one death (previously unrecognized silent MI), ventricular tachycardia (*n* = 2) requiring cardioversion, and respiratory infection (*n* = 2). LVEF in the ischemic subgroup (*n* = 41) improved by 4.7% ± 9.7 from pre-procedure to the first follow-up (4 months ± 1.9 months) (*p* < 0.004) and by 7.2% ± 10.9 at final follow-up (*n* = 25) at average 12 months (*p* < 0.004). The non-ischemic dilated cardiomyopathy subgroup (*n* = 8) improved by 7.5% ± 6.0 at the first follow-up (*p* < 0.017) and by 12.2% ± 6.4 at final follow-up (*p* < 0.003, *n* = 6). Overall improvement in LVEF from pre-procedure to post-procedure was significant (Fisher’s exact test *p* < 0.004). LVEF improvement was most marked in the patients with the most severe cardiomyopathy (LVEF < 20%) improving from a mean 14.6% ± 3.4% pre-procedurally to 28.4% ± 8% at final follow-up. Quality of life statements reflected improvement in 33/50 (66%), no change in 14/50 (28%), and worse in 3/50 (6%).

**Conclusion:**

Transcatheter implantation of ACP-01 for cardiomyopathy is safe and improves LVEF in the setting of ischemic and non-ischemic cardiomyopathy. The results warrant further investigation in a prospective, blinded, and controlled clinical study.

*Trial Registration*: IRB from Genetic Alliance #APC01-001, approval date July 25, 2022.

**Condensed Abstract:**

Cardiomyopathy is common and associated with high mortality. Stem cell transplantation is an emerging therapy. Angiogenic cell precursors (ACP-01) are lineage-specific endothelial progenitors, with strong potential for migration, engraftment, angiogenesis, and support of tissue survival and regeneration. A retrospective outcomes analysis of 53 patients with ischemic and non-ischemic dilated cardiomyopathy undergoing transcatheter implantation of ACP-01 demonstrated improvements in the left ventricular ejection fraction of 7.2% ± 10.9 (*p* < 0.004) and 12.2% ± 6.4, respectively, at 12 months (± 5) follow-up. Quality of life statements reflected improvement in 33/50 (66%) patients.

## Introduction

The incidence of heart failure within the USA is 1.6%, of whom 42.3% will die within 5 years of hospitalization [1, 2]. The Centers for Disease Control and Prevention reports the incidence of heart failure in the USA at 6.3 million adults [1]. Heart failure may be caused by a wide range of abnormalities of cardiac structure and function. Central to the evaluation and management of heart failure is the left ventricular ejection fraction (LVEF)—a core measure of cardiac efficiency. This series of heart failure patients in whom medical management had failed presented with reduced LVEF due to ischemic cardiomyopathy or non-ischemic dilated cardiomyopathy. In the former, ischemia results in thinning and scarring of the cardiac muscle. In the latter, a variety of insults result in dilation and remodeling of the left ventricle, manifesting in chest pain, dyspnea, edema, cardiac arrhythmia, and death.

An emerging therapy for heart failure is stem cell transplantation. Stem cells secrete growth factors, stimulate angiogenesis, replace damaged cells, and exhibit an anti-inflammatory effect to minimize scarring around ischemic tissue [3-6]. Sources of stem cells used for the treatment of myocardial ischemia include autologous bone marrow mononuclear cells (BMNCs), bone marrow-derived mesenchymal stem cells (MSCs), peripheral blood mononuclear cells (PBMCs), skeletal myoblasts, cardiac progenitor/stem cells, human embryonic stem cell-derived cardiomyocytes, and human-induced pluripotent stem cell-derived cardiomyocytes (hiPSC-CMs) [7].

The angiogenic cell precursors (ACP-01), in this study, are autologous, isolated from adult peripheral blood, derived from a multipotent progenitor cell population, and designated a synergetic cell population (SCP) [5]. ACP-01 secrete angiogenic factors, including vascular endothelial growth factor (VEGF), angiogenin, and the cytokine interleukin-8 (CXCL8) [5], which is known for its stem cell recruitment, anti-apoptotic capabilities, and proangiogenic capabilities [8].

ACP-01 support tissue survival and regeneration and possess the characteristics of lineage-specific (that is, specifically programmed) endothelial progenitors, demonstrating strong potential for effective engraftment and blood vessel formation [5]. Preclinical and clinical studies in which intramyocardial injections of ACP-01 were delivered thoracoscopically have demonstrated improved cardiac function [9, 10]. The present outcome analysis of patients with ischemic and non-ischemic dilated cardiomyopathy is the first to demonstrate significant efficacy following implantation of ACP-01 by transcatheter, intramyocardial, and/or intracoronary techniques.

## Methods

This is a retrospective outcome analysis of 74 consecutive patients who underwent intramyocardial or intracoronary injection of autologous ACP-01 in Thailand [5 patients] and in the Dominican Republic (69 patients, 2008–2012). Separate and detailed approvals were collected, and informed consent obtained for the administration of this treatment. The treatment was performed within a compassionate use protocol. This retrospective analysis received IRB approval. The inclusion/exclusion criteria for the compassionate use protocol (2005–2012) are stated below.

## Inclusion criteria

- Diagnosis of ischemic and dilated non-ischemic cardiomyopathy
- Angina (Classes II–IV)
- Receiving maximal medical therapy, defined as a medical regimen that includes the maximal tolerated dose of at least two antianginal medications, such as beta-blockers, nitrates, or calcium channel blockers
- Hemodynamic stability

## Exclusion criteria

- Unstable angina
- Heart transplant
- Abnormal anatomy, severe valvular disease, or mechanical aortic valve that preclude safe entry of the catheter into the left ventricle
- Pregnancy and lactation
- Coagulopathy
- Left ventricular ejection fraction (LVEF) ≥ 50%

### Data collection for the retrospective analysis

Patients were de-identified and given a study ID number for data collection. The following data points were retrieved from the patient charts (Table 1):

- Diagnosis: type of cardiomyopathy
- Age
- Means of delivering the ACP-01
- Cell count positive for CD31^+^, CD34^+^; viability and number of cells injected into each... patient.
- Date and location of treatment
- Pre-procedural and post-procedural LVEF measured by multi-gated acquisition scan (MUGA), echocardiogram, or single-photon emission computerized tomography (SPECT)
- Comorbid conditions
- Complications: peri-procedural and within the 3-month follow-up period
- Subjective patient-reported assessments as to quality of life at 6 months (± 5.6 months)

**Table 1:** Characteristics of patients meeting criteria for data analysis

ID #	Sex	Age at Tx	Dx*	Comorbidities/PMH	Pretx LVEF%	Posttx LVEF% #1	Test interval #1 (mo)	Posttx LVEF %#2	Test interval #2 (mo)	QOL	Injection method	Comments CD34/CD31 × 10^6^
1	m	63	I	Aortic valvoplasty, CABG, and aortic aneurysm	48 (M)	35 (E)	3			Better	IC	2nd tx 7.6/8.1
3	m	54	I	CAD, MI, and ICD	21 (E)	23 (E)	5	33 (E)	11	Same	LV	13.7/49.1
8	f	21	N	23 (M)	37 (E)	3.5	37 (E)	15.5		Worse	???	3 0/19.4
9	m	65	I	CAD, CABG, Vtach, and ICD	11 (E)	20 (E)	9			Same	???	3.5/14.7
10	m	77	I	LV thrombosis, CAD, MI, Prim PCI, and pacemaker	36 (E)	38 (E)	3.5			Better	LV	3.3/7.9
12	m	60	N	Pacemaker, CAD, and MI	17 (M)	30 (E)	4	40 (E)	6	Better	LV	1.7/4.2
13	m	71	I	Pacemaker, CAD, and CABG	31 (M)	55 (E)	3	37 (M)	7	Same	???	5.9/14.9
15	m	75	I	CAD, CVA, aphasia, vertigo, and CABG	19 (M)	27.5 (E)	1.5			Worse	???	
16	m	69	I	CABG and pacemaker	45 (E)	30(E)	6			Same	LV	2nd tx 2.4/44.3
18	m	79	I	Pacemaker, defib, CAD, CABG, and angioplasty	50 (E)	45 (M)	3	50 (M)	6.5	Better	???	6.3/7.4
19	m	79	I	Defib, Mx2, pacemaker, and angioplasty	36 (M)	41 (M)	3	49 (M)	12	Better	???	13.7/9.9
20	m	52	N	Testicular carcinoma	25 (E)	29 (M)	5	40 (E)	8.5	Better	LV	1.1/11.1
23	m	71	I	CABG, ICD, AAA, and COPD	26 (M)	30 (E)	1.5	20 (E)	5	Same	LV	1/3
24	m	74	^1^	MV repair(ring), testicular Ca, prostate Ca, pacemaker, angioplasty, and MI	27.5 (E)	26 (E)	4			Better	???	4.3/33.6
25	m	57	I	CAD, MI, and angioplasty	48 (E)	62(E)	4.5	79 (E)	25.5	Same	???	45.7/35.1
26 27	m m	59 63	CHF	ICD, DM, colon cancer MI × 4, angioplasty × 4, ICD, Vtach, and cardiac arrest	17.5 (E) 14 (M)	25 (E) 15 (E)	3 3.5	27.5 (NST)	12.5	Better ???	LV LV	2.3/1.0 3.2/21.4
30	m	52	I	MI, angioplasty, CABG, and DM	27.5 (E)	50 (E)	5	53 (S)	17	Better	???	2.1/10.4
31	m	70	I	MI, Afib, defib, and angioplasty	14 (M)	15 (E)	3.5			Same	IC/LV	3.0/35.5
32 33	m m	54 70		DM, CVA, dysphagia, MI, Afib, ICD, angioplasty, sleep apnea, CPAP MV repair, CABG, CAD, and mod–sev AS MR	27.5 (E) 24 (M)	27 (M) 31 (M)	4.5 *A*	22.5 (E) 25 (M)	8.5 12	Same Better	LV ???	5.3/10.5 15.3/17.5
34	f	50	N	LV epicardial lead placement repair of LV tear, ICD, COPD, morbid obesity, and DM	11 (E)	10 (E)	3.5			???	LV	11.7/23.3
35	m	74	I	CAD, MI X2, angioplasty, and DVT	34 (M)	41 (M)	3	45 (M)	12	Better	LV	3.1/15.3
36	m	70	I	CAD, MI, CABG, VSD repair, and Afib	38 (S)	40 (E)	7 5	45 (E)	13	Better	???	2.6/25.0
38	f	65	I	LV aneurysm and angioplasty	29 (M)	22.5 (E)	7.5			Better	LV	4.6/18.5
39	m	66	N	ICD	43 (M)	45 (M)	3	50 (M)	15.5	Better	LV	4.6/33.3
40	m	74	I	CABG, Afib, ablation, and ICD	28 (M)	37 (M)	8			Better	???	8.5/9.7
41	m	73	I	MI, CABG, and ICD	43 (M)	33 (E)	8	49 (M)	27	Better	???	2ndtx 23.5/15.4
42	f	66	CHF	Metastatic breast Ca (bone) and hypothyroidism	45 (E)	25 (E)	4	20 (E)	8	Better	IC/LV	4.5/10.9
43	m	66	I	MI, angioplasty, and ICD	33 (M)	45 (M)	3	25 (M)	8	Better	???	1.5/13.5
44	m	83	I	CAD, DM, RA, CABG, and pacemaker	31 (M)	35 (E)	1.5	25 (M)	10	Better	LV	2nd tx 6.3/10.0
45	m	79	^1^	MI, CABG, DM, PAD stent, ICA occlusion, endarterectomy, hypothyroidism, and dementia	42 (M)	67 (E)	1			Better	LV	10.1/15.7
46	m	77	?	Reduced LV size and new viable myocardium	30 (E)	37 (M)	4	41 (M)	23	Same	???	lstof2tx 1.7/3.4
47	m	72	^1^	CAD, MI, angioplasty, CABG, Afib, ICD, DM, carotid stent, and CPAP	38 (M)	50 (M)	3	51 (M)	9.5	Better	???	2.2/13.11
49	m	74	I	Angioplasty	23 (M)	38 (M)	3	35 (M)	24	Better	LV	13.3/13.1
50	m	71	^?^	CAD, MI, and pacemaker/defib	8 (< M)	8.4 (CM)	3	22.5 (E)	13.5	Better	IC/LV	9.0/32.8
51	m	68	I	MI, angioplasty, CABG, ICD, and skin Ca	30 (M)	36 (M)	3.5	46 (M)	12	Better	LV	4.3/15.6
52	m	68	I	MVR and pacemaker	35 (M)	43 (M)	4	21 (M)	10	Better	???	3.1/18.6
53	m	82	I	CAD, MI, CABG, and pacemaker/defib	21 (M)	24 (M)	3	29 (M)	12	Same	???	19.3/32.8
55	m	79	^1^	MIx2, angioplasty, ICD, and thoracotomy for experimental gene tx	36 (E)	22.5 (E)	8.5			Same	LV	3rd tx 7.7/28.4
57	m	68	I	Viral cardiomyopathy	20 (M)	30(E)	1.5			???	???	21.1/29.4
59	m	77	I	CAD, Afib, pacemaker/ICD, and CABG	22 (M)	35 (M)	6.5	41 (M)	14.5	Better	???	6.5/18.2
60	m	61	N	Fabry disease, pacemaker/ICD, ESRD, dialysis, COPD, hyperthyroidism, and radioactive ablation	28 (M)	41 (M)	4	40 (M)	12.5	Better	???	13.7/40.9
61	m	77	^1^	MIx2, CABG, Afib/flutter, Vtach, pacemaker/ICD, and hypothyroidism	15 (E)	32 (M)	4.5	32 (M)	12	Better	LV	2.0/13.3
62	m	78	^1^	CAD, MI, CABG, and pacemaker/defib	31 (M)	33 (M)	3.5	35 (M)	7.5	Better	LV	lstof2tx 4.8/41.0
64	m	46	^1^	ESRD, renal transplantx2, dialysis, pancreas transplant(failed?), DM I, and pacemaker	17 (M)	25(E)	1.5			Worse	???	4.9/19.2
65	m	74	I	MI, CABGx3, and ICD	28 (M)	30(E)	2	15 (E)	6.5	Better	LV	18.2/88.5
66 68	m m	81 58	I I	CAD, multiple angioplasties, CABG, DM, Afib, gene therapy EECP CAD, MI, angioplasty, ICD/pacemaker, decompensated CHF, hypothyroidism, and morbid obesity	37 (M) 33 (M)	49 (M) 37 (M)	4.5 3.5	36 (S)	15	Same Better	??? LV	8.7/21.5 2.9/5.1
69	f	73	N	ICD/pacemaker	17.5 (E)	25 (E)	4	20 (E)	12	Same	LV	17.1/29.8
70	m	65	N	Renal insufficiency	43 (E)	55 (E)	19			Better	IC/LV	6.7/40
*TT*	m	68	I	CABG and Afib	22.5 (E)	25 (E)	4	34 (M)	7	Better	LV	4.4/10.7
73	m	81	I	CAD, angioplasty, MI, Afib, and ICD	32.5 (E)	30 (E)	4			Same	LV	4.0/34.4

The patients are listed by ID number. Only patients undergoing statistical analysis are included in the table

### Preparation of the ACP-01 for transplantation and flow cytometry

Autologous blood was obtained by simple peripheral blood draw (250 mL) and transported to the manufacturing facility. The preparation and characterization of the ACP-01 is described in detail elsewhere [5, 9]. Briefly, a certain fraction of peripheral blood mononuclear cells, the synergetic cell population (SCP), was isolated and cultured for 5 days under conditions that induce their differentiation into ACP-01 [5]. 5 × 10^5^ cells per sample were stained; at least 10^4^ cellular events per sample were assessed by flow cytometry and analyzed by CellQuest Pro software (Becton Dickinson).

**Fig. 1:**
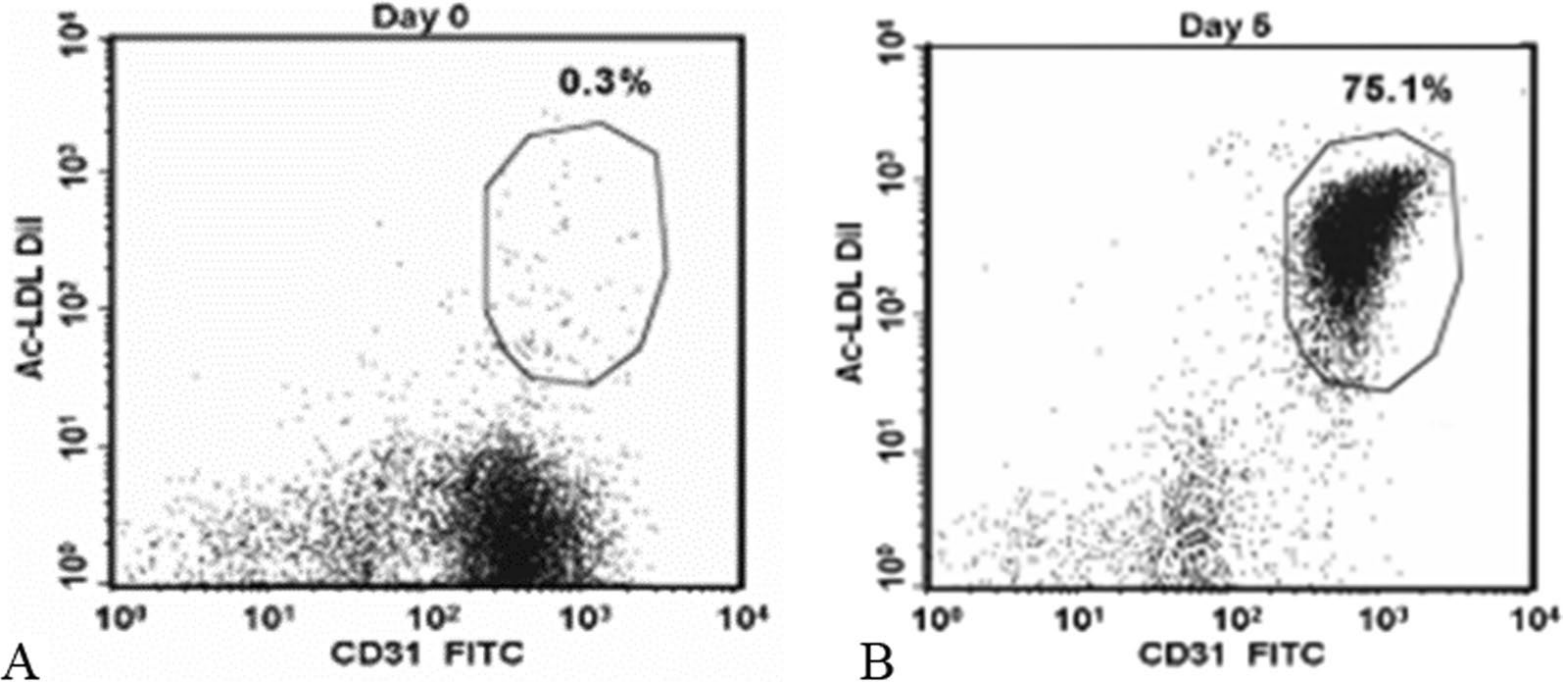
Flow cytometry analysis of ACP-01. Flow cytometry analysis of ACP-01 demonstrating initial expression of anti-CD31 + FITC before culture (**A**) and concomitant increased uptake of Ac-LDL after incubation (**B**).

The ACP-01 were analyzed for expression of CD34^+^, CD133^+^, and CD117^+^ markers typical of multipotent hematopoietic stem cells (HSCs), as well as the KDR, Tie2, CD144^+^, vWF, and CD31^+^ endothelial cell markers. The CD31^+^ and CD34^+^ were the percentage of cells with bright intensity CD31^+^ bright and CD34^+^ bright, respectively, where staining intensity was at least 50 times higher than the intensity of control staining. For acetyl low-density lipoprotein (Ac-LDL) uptake, cells were incubated in the presence of Ac-LDL (Alexa Fluor488 Ac-LDL or Ac-LDL-DiI) and stained with FITC- or PE-conjugated CD31^+^ (Fig. 1)**.**

### Tube formation assay

**Fig. 2:**
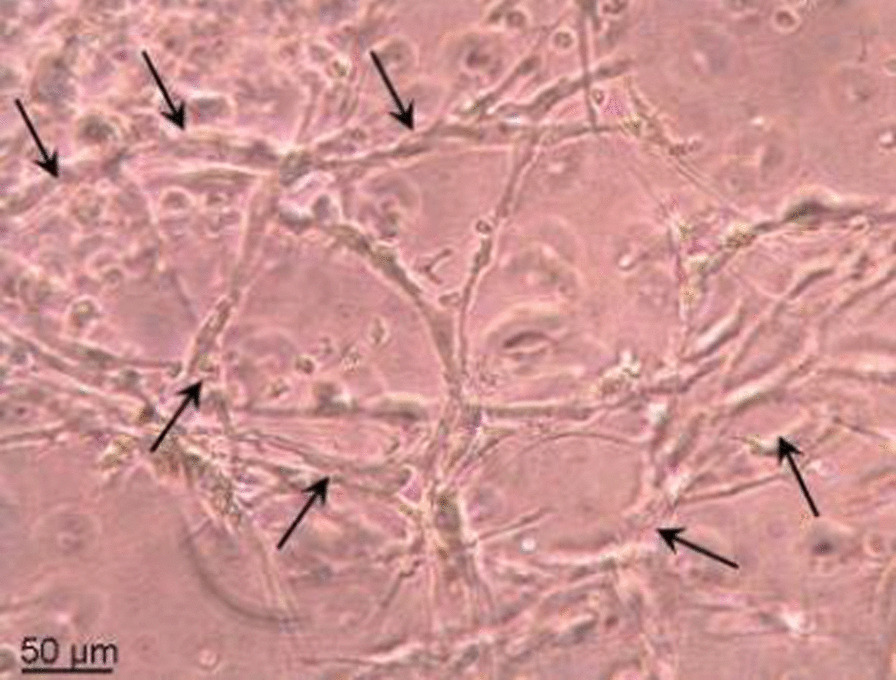
Tube formation assay. The arrows indicate cells, programmed to form endothelial cells, organizing into tube-like structures

Tube formation was tested using an in vitro angiogenesis assay kit. Harvested ACP-01 were cultured overnight in 10% autologous serum, with 10-ng/ml VEGF, 10-ng/ml basic fibroblast growth factor (bFGf), 5-IU/ml heparin, and 25-ng/ml endothelial cell growth supplement on extracellular matrix (ECM) gel. Tube formation was assessed visually using an inverted light microscope. Angiogenic pattern and vascular tube formation were demonstrated: The ACP-01 cells organized into tube-like structures, which are programmed to form endothelial cells (Fig. 2).

Similar to endothelial progenitor cells, ACP-01 display uptake of Ac-LDL and secrete tissue regeneration factors such as CXCL-8, VEGF, and angiogenin. Following this differentiation stage, the ACP-01 were harvested, packed in syringes, transported to the hospital, and administered to patients.

### Transcatheter injection technique

Within 1 week of blood collection, the ACP-01 were injected by a transcatheter technique into the diseased myocardium or coronary arteries of patients under local anesthesia (Central Illustration). Cell injection was carried out using SR 200 MyoCath® 25-gauge transmyocardial catheter via femoral artery access. The thickness of the LV wall, predetermined by echocardiogram, determined the depth of injection. The technique of transcatheter intramyocardial and intracoronary injection is elaborated elsewhere [11, 12].

### Data analysis

The pre-procedure and post-procedure LVEFs were treated as continuous variables, expressed as probability density functions, and presented as mean ± standard deviation. The LVEF data fulfilled the assumption for parametric testing (namely, normal distribution); therefore, the paired t-test was used to compare mean preoperative and postoperative LVEF. A p-value of < 0.05 was considered significant. The patients were divided into four quartiles based upon pre-procedure ejection fraction, and a Fisher’s exact test was used to assess statistical likelihood of a patient moving up to the next one or two categories after ACP-01 treatment.

### Outcome measures for safety

Safety endpoints included incidence of treatment-related serious adverse events (SAEs) in relation to the treatment procedure and in the follow-up period. SAEs would include events leading to hospitalization, death, significant disability, MI, stroke, hospitalization for worsening heart failure, cardiac perforation, pericardial tamponade, sustained ventricular arrhythmias, and new-onset atrial fibrillation.

### Quality of life assessment

Quality of life statements were subjective statements offered by the patient, gleaned from the office notes, and analyzed as a categorical variable. No standardized quality of life instrument or scale was administered at the time of treatment. The statements recorded were retrospectively aggregated and subjected to a 3-point scale, independently by two authors, based upon whether the patient was: 1. improved and expressed benefit from the procedure; 2. neutral as to whether the procedure was helpful, or 3. worse after the procedure. These data were treated as ordinal data and underwent a Chi-square analysis. Non-parametric statistical hypothesis testing with the Chi-square test was used for the 3-point scale, given that there is no established distribution of the effect of patient symptomatic improvement following the injection of ACP-01. The results of the patients diagnosed with ischemic cardiomyopathy were compared with non-ischemic cardiomyopathy by simple comparison of mean changes pre- and post-procedure and a two-tailed Student’s t-test, accepting a significance of alpha of 0.05.

## Results

**Fig. 3:**
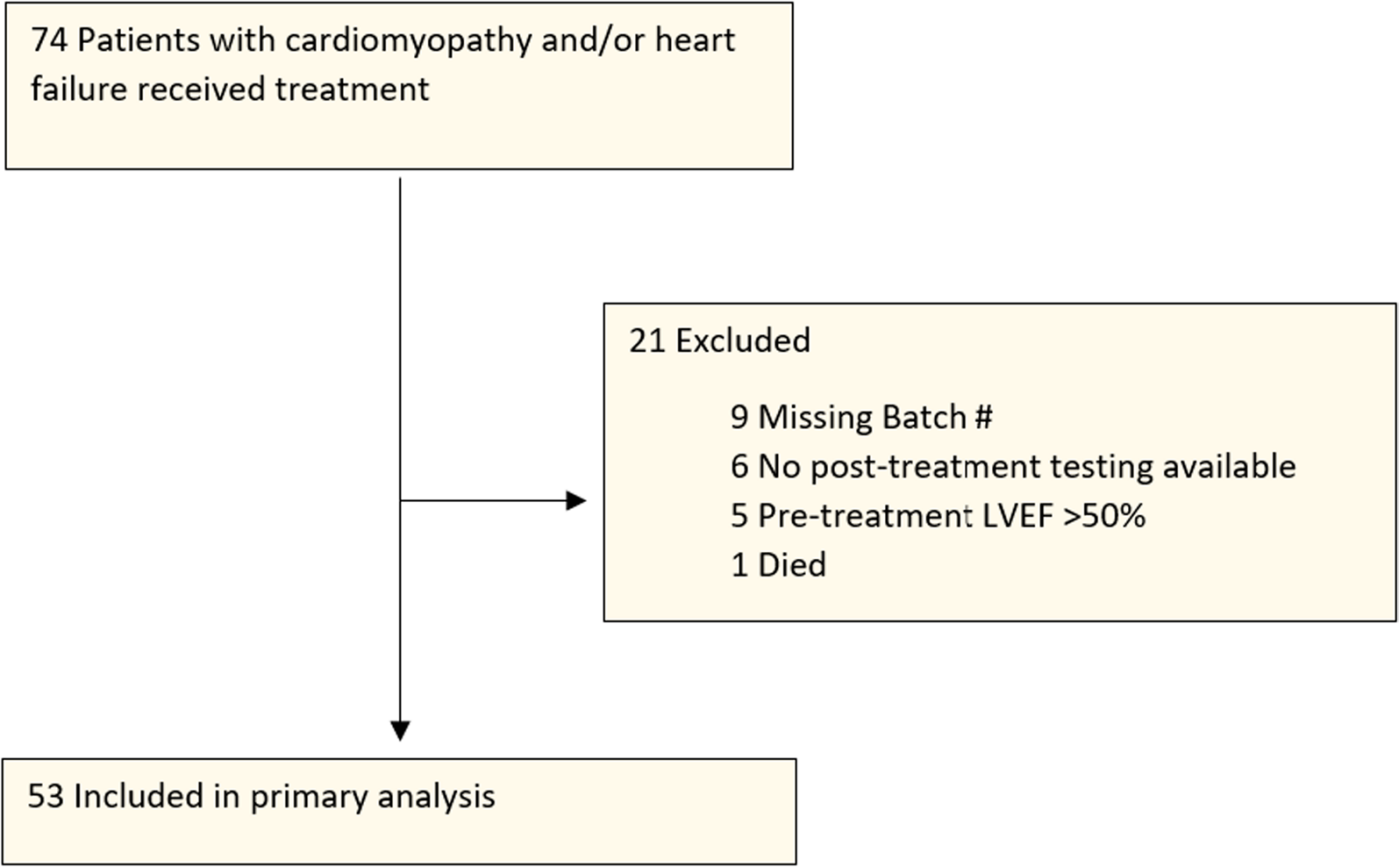
Flow diagram. Of the 74 consecutive patients undergoing review, 21 were excluded for incomplete documentation or LVEF > 50%.

Of the 74 consecutive adult patients who underwent the implantation of ACP-01 for the treatment of cardiomyopathy, 21 were excluded from analysis on the basis that pre- or post-procedure LVEFs were not recorded in the chart, certification of cell analysis documentation of stem cell therapy was incomplete, or preoperative LVEF was > 50% (patients with normal LVEF who had been treated for intractable angina). One patient died within 1 day of implantation procedure (flow diagram, Fig. 3). Fifty-three patients underwent analysis: 48 males and five females; age 68.1 ± 11.3 years (Table 1). The severity of disease and preponderance of comorbidities are noted (Table 2).

**Table 2:** Comorbidities in cardiomyopathy subjects

Variable	% subjects (*n* = 53)	Variables cont'd	% subjects (*n* = 53)
Age (years)	68.1 ± 11.3	ICA occlusion	1 (2%)
Male	90.6%	LV aneurysm	1 (2%)
*Cardiac*		LV thrombosis	1 (2%)
Myocardial infarction	25 (47%)	PAD stent	1 (2%)
CABG	23 (43%)	*Renal*	
ICD	21 (40%)	ESRD	2 (4%)
Coronary artery disease	20 (38%)	Dialysis	2 (4%)
Pacemaker	18 (34%)	Renal insufficiency	1 (2%)
Angioplasty	18 (34%)	*Other*	
Atrial fibrillation	10(19%)	Hypothyroidism	4 (8%)
Defibrillation	6(11%)	COPD	3 (6%)
Ventricular tachycardia	3 (6%)	CPAP	2 (4%)
Mitral valve repair	3 (6%)	Morbid obesity	2 (4%)
Ablation	1 (2%)	Testicular Ca	2 (4%)
Cardiac arrest	1 (2%)	Aphasia	1 (2%)
Decompensated CHF	1 (2%)	Colon carcinoma	1 (2%)
Left ventricle tear	1 (2%)	Dysphagia	1 (2%)
Primary PCI	1 (2%)	Metastatic breast Ca	1 (2%)
Reduced LV size	1 (2%)	Mod–sev AS MR	1 (2%)
Viral cardiomyopathy	1 (2%)	Pancreas transplant	1 (2%)
Aortic valvuloplasty	1 (2%)	Prostate carcinoma	1 (2%)
Carotid stent	1 (2%)	RA	1 (2%)
*Other vascular*		Renal transplant	1 (2%)
*AAA*	1 (2%)	Sleep apnea	1 (2%)
Aortic aneurysm	1 (2%)	Vertigo	1 (2%)
Cerebrovascular accident	2 (4%)	VSD repair	1 (2%)
Diabetes mellitus	9(17%)	Dementia	1 (2%)
Deep vein thrombosis	1 (2%)	Skin cancer	1 (2%)
EECP	1 (2%)	Fabry disease with	1 (2%)
Endarterectomy	1 (2%)	Hyperthyroidism	

AAA = abdominal aortic aneurysm, AS = aortic stenosis, CA = cancer, CABG = coronary artery bypass graft, CHF = congestive heart failure, COPD = chronic obstructive pulmonary disease, CPAP = continuous positive airway pressure, DVT = deep vein thrombosis, EECP = enhanced external counterpulsation, ESRD = end-stage renal disease, ICA = internal carotid artery ICD = implantable cardioverter defibrillator, LV = left ventricle, MR = mitral regurgitation, PCI = percutaneous catheter intervention, RA = rheumatoid arthritis, and VSD = ventricular septal defect

### Pre-procedure and post-procedure LVEF

The first follow-up LVEF of all patients after implantation of ACP-01 (*n* = 52) revealed a significant improvement of LVEF of 4.6% (*p* < 0.001) (Table 3). Comparing the pre-procedure LVEF of all the patients with the final follow-up at 12 months, the mean improvement in LVEF was 7.7% (*p* < 0.003). In the analytic dataset of the 53 patients, 52 had LVEF at the first follow-up (4 months ± 1.9 months), but one did not have the 4-month LVEF and was, therefore, not in the analysis of the initial follow-up; this patient, however, did have a LVEF for the final follow-up (12 months ± 5.5 months) and is included in the final follow-up data. Fewer observations (*n* = 35) were available for the final follow-up (Table 4).

**Table 3:** Overall LVEF of all patients pre-procedure and at the first follow-up (4 months ± 1.9 months after procedure), demonstrating improvement

Variable	*n*	Mean	SD	95% conf. interval
Pre-treatment	52	28.6	10.4	25.7–31.5
Post-treatment	52	33.2	12.0	29.9–36.6
Difference	52	4.6	9.6	− 7.3– − 1.9
*t* = − 3.4607				
*p* < 0.001				

Paired *t*-test

**Table 4:** Overall LVEF of all patients pre-procedure compared with final follow-up (average 12 months ± 5.5 months) showing significant improvement

Variable	*n*	Mean LVEF	SD	95% conf. interval
Pre-treatment	35	29.8	10.0	26.3–33.2
12-month follow-up	35	37.4	12.9	33.0–41.9
Difference	35	7.7	11.2	− 11.5–3.9
*t* = − 4.08				
*P* < 0.003				

Paired t-test

### Outcomes of ischemic versus non-ischemic cardiomyopathy

**Fig. 4:**
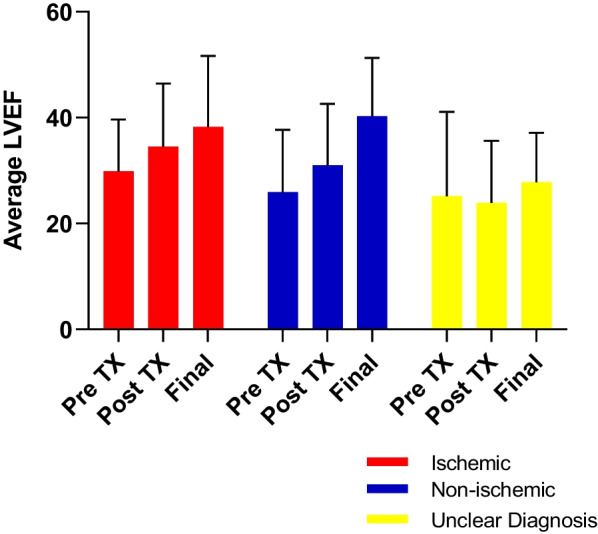
LVEF pre-procedure and post-procedure at 4 months and at final follow-up; patients grouped by diagnosis. The mean LVEFs are given for subjects with ischemic cardiomyopathy (red) and non-ischemic dilated cardiomyopathy (blue) and four patients in whom it was unclear whether the cardiomyopathy was ischemic or non-ischemic (yellow). The values are treated as continuous variables. Paired t-test demonstrated significant improvement at 4 months (post-treatment) and at 12 months in the ischemic and non-ischemic categories; there was no significant improvement in the patients for whom the diagnosis was unclear

The ischemic subgroup (*n* = 41) improved by 4.7% ± 9.7 from pre-procedure to post-procedure (4 months ± 1.9 months) (*p* < 0.004) and by 7.2% ± 10.9 from pre-procedure to final follow-up (*p* < 0.004, *n* = 25). The non-ischemic dilated cardiomyopathy subgroup (*n* = 8) improved by 7.5% ± 6.0 from pre-procedure to post-procedure at 4 months (*p* < 0.017) and by 12.2% ± 6.4 from pre-treatment to final follow-up (*p* < 0.003, *n* = 6). Four patients with cardiomyopathy did not clearly fall into the ischemic or non-ischemic categories: Two of these exhibited a 3.7% improvement at the first follow-up and 12.8% improvement at final follow-up; the other two patients demonstrated worsening of ejection fraction (− 6.3% at the first follow-up and − 7.5% at final follow-up). These latter four patients are grouped as the “unclear” diagnosis (Fig. 4).

### Improvement according to quartile of severity

**Fig. 5:**
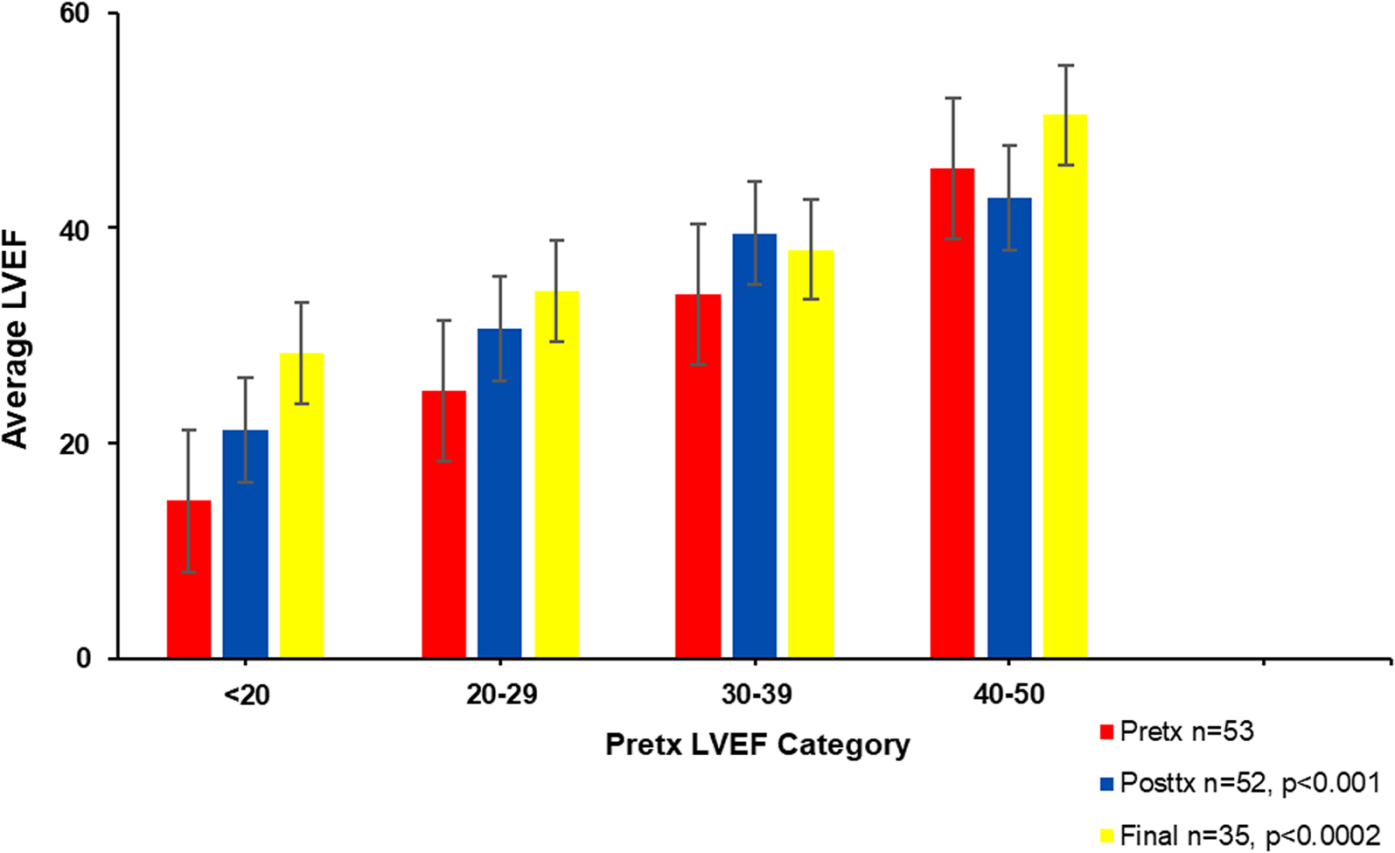
LVEF improvement according to quartile of severity. Pre-procedural LVEF (red), compared with 1st set of post-procedure LVEF measurements (blue) at 4 months ± 1.9 months (range 1–9 mo) and final follow-up (yellow). The LVEFs were measured by MUGA, echocardiogram, or SPECT; the values were treated as continuous variables and compared by paired t-test which demonstrated a significant improvement from pre-procedure to final follow-up (*p* < 0.0002). Overall, the improvement in LVEF from pre-procedure to post-procedure was statistically significant (Fisher’s exact test *p* < 0.004). The improvement was most marked in the patients with the most severe cardiomyopathy (LVEF < 20%)

To determine which quartile of severity showed the most benefit from the cell implantation, the patients were divided according to pre-procedure LVEF:

(i) LVEF < 20; (ii) LVEF 20–29; (iii) LVEF 30–39; and (iv) LVEF 40–50. All four groups improved, three moving upward into the next quartile of LEVF (Fig. 5). Overall, upward mobility of LVEF was significant for the first follow-up (*p* < 0.001) and for the final follow-up (*p* < 0.0002). The improvement was most marked in the patients with the most severe cardiomyopathy (LVEF < 20%) improving from a mean pre-procedural LVEF of 14.6% ± 3.4% to 28.4% ± 8.0% at final follow-up. LVEF in patients in the next most severe category (LVEF 20–29%) improved from 24.9% ± 3.0% to 34.1% ± 9.2% at final follow-up. For the patients in the 30–39% category, LVEF improved from 33.8% ± 2.8 to 38% ± 10.0 at final follow-up. Finally, in the 40–50 category, LVEF improved from 45.5% ± 2.8 to 50.5% ± 18.8%.

### Power analysis

A post hoc power analysis demonstrated that the study had 89% power to detect the observed LVEF pre- and post-procedure difference of 5%. In designing a future study, the estimated sample size to detect a hypothesized significant difference of LEVF would be 31 patients, assuming that the sample parameters (i.e., variability) are the same as observed in this study.

### Adverse reactions

**Fig. 6:**
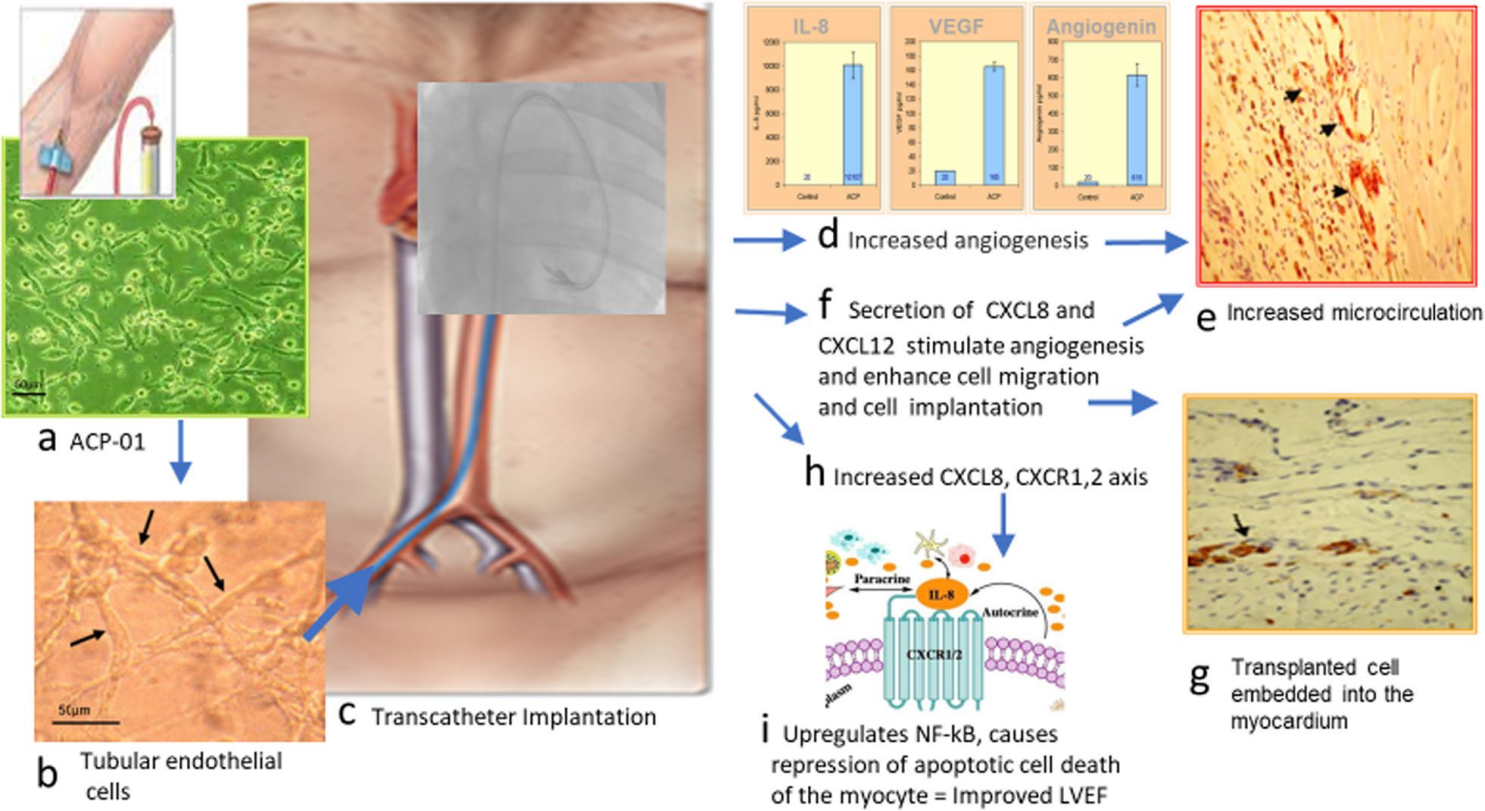
Central Illustration: transcatheter implantation of ACP-01 for cardiomyopathy. Autologous multipotent hematopoietic cells obtained by a blood draw undergo a process of trans-differentiation to ACP-01 **a** which are programmed to form tubular, endothelial cells (**b**). Cells undergo transcatheter, intramyocardial, or intracoronary implantation of ACP-01 (**c**). Cytokine expression of CXCL8, VEGF, and angiogenin (**d**) promotes angiogenesis (**e**). Small black arrows indicate neo-angiogenesis (photomicrograph X400). The expression of CXCL8 (**f**) enhances cell migration and cell implantation (**g**). Injured myocardium expresses CXCL12 ligand (**f**) which attracts the CXCR 4 receptors on ACP-01, enhancing migration (**g**) to the ischemic or non-ischemic dilated myocardium (photomicrograph X400). Elevated CXCL8/CXCR1,2 axis (**h**) expressed by ACP-01 repress apoptosis through upregulation of the NF-kB axis (**i**). These function collectively to improve LVEF

One patient died within 24 h of cell injection due to the left ventricular wall perforation, attributed to faulty catheterization in a patient who had suffered an unrecognized silent myocardial ischemia event 1 month before. Two patients developed ventricular tachycardia requiring electrical cardioversion. One patient was hospitalized for pneumonia 6 days after one treatment and another for a bacterial respiratory infection 14 days after the procedure. There were no known injuries to target vessels, no heart failure hospitalizations, and no re-infarction. There were no pericardial tamponade, no sustained ventricular arrhythmia, or new-onset atrial fibrillation (Fig. 6).

### Quality of life statements

Fifty patients contributed quality of life statements following the procedure. These were grouped: improved = 33/50 (66%), no change = 14/50 (28%), or worse = 3/50 (6%). Within each quality of life category, comparison of the difference in the pre-procedure and post-procedure, and pre-procedure and final follow-up mean LVEF results did not detect statistically significant differences (*p* < 0.5 and *p* < 0.3, respectively). Changes in pre-procedure to post-procedure outcomes by LVEF categories were compared to the categorized quality of life statements (better, no change, and worse). This comparison did not reach statistical significance (Fisher’s exact test, *p* < 0.9).

### Delivery method

The delivery method was documented for 29 of the 53 patients as follows: left ventricular (LV) intramyocardial = 24, intracoronary/LV intramyocardial = 4, and intracoronary = 1. Documentation was unclear as to the method of delivery of cells in the remaining 24 patients. Comparison of the pre-procedure and final follow-up LVEF by delivery method did not detect statistically significant differences (Fisher’s exact *p* < 0.9).

### Characteristics of the injected cells

The number and viability of cells were tested by trypan blue exclusion. The cell batch passed quality control before use. The total number of viable cells was 57.4 ± 4.1 × 10^6^ with cell viability ≥ 97% ± 2.4. Each batch was certified negative for bacterial culture, gram stain, mycoplasma, and endotoxins. The number of cells expressing CD34^+^ was 7.7 × 10^6^ ± 7.8 per batch, and the number of cells expressing CD31^+^ was 21.1 ± 15.3 × 10^6^ or 39% of total cell number. The cells expressed high levels of CXCL8, as measured by ELISA (Table 5).

**Table 5:** Characteristics of injected cell batches

Characteristics	ACPs
Total cells/product (× 10^6^), mean (SE)	57.4 (4.1)
% viability/product by trypan blue exclusion	
Mean (SE)	97.6 (0.5)
Median (range)	97.8 (90–100)
Number of CD34^+^ cells/product (× 10^6^), mean (SE)	7.7 (1.1)
Number of CD31^+^ cells/ product (× 10^6^), mean (SE)	21.1 (2.1)
% ACP cells/product, mean (SE)	39.1 (3.2)
IL-8 ng/dose, mean (SE)	647.4 (193.0)

*Standard error (SE)

## Discussion

### ACP-01 implantation was associated with robust and durable improvement in LVEF

This is the first outcomes analysis to demonstrate the beneficial effects of transcatheter intramyocardial/intracoronary implantation of ACP-01. Significant overall improvement of LVEF was demonstrated post-procedure (4.6%; *p* < 0.001) and further improvement at final follow-up with an overall increase in LVEF of 7.7% (*p* < 0.003). The outcomes for ischemic cardiomyopathy were inferior to those with non–ischemic dilated cardiomyopathy at 4 months and at final follow-up, wherein the ischemic cardiomyopathy group demonstrated increase of 7.2% LVEF, compared to the non-ischemic group in whom an improvement in LVEF of 12.2% was demonstrated. While those patients with dilated non-ischemic cardiomyopathy appear to have shown the greatest benefit from ACP-01, the difference was not statistically significant. Of the four patient quartiles based upon severity of cardiomyopathy in terms of LVEF, three improved into the next higher quartile of LVEF. Patients in the most severe category of cardiomyopathy (LVEF < 20%) demonstrated the greatest upward mobility, suggesting that these patients with the most severe disease would most likely to significantly benefit from ACP-01. Overall upward mobility was significant for the first follow-up (*p* < 0.001) and for the final follow-up (*p* < 0.0002). The quality of life statements made by the patients after treatment suggested overall improvement in most (66%); but this improvement did not reach statistical significance, because several patients reported improved function and quality of life despite a decline in LVEF. The latter was attributed to the reporting of the quality of life at different times to the measurement of LVEF.

The transcatheter application of ACP-01 for cardiomyopathy appears *prima facie* to compare favorably against controlled, double-blinded studies that utilized bone marrow-derived stem cells, and adipose-derived MSCs. The late TIME trial, of 87 patients enrolled through the Cardiovascular Cell Therapy Research Network (CCTRN) who underwent transcoronary injection of autologous BMNC 2–3 weeks after anterior MI, demonstrated no difference in LVEF or wall motion in the infarct zone [13]. Similarly, the use of autologous BMNC demonstrated no improvement in primary and secondary outcomes in the FOCUS–CCTRN trial (*n* = 92), the REGENERATE-AMI phase II study (*n* = 100) treating severe ischemic cardiomyopathy, or the MIHEART multicenter trial treating non-ischemic dilated cardiomyopathy [14-16]. The use of autologous adipose-derived MSC injected intramyocardially in the Athena trial similarly showed no improvement in LVEF [17].

Positive results with allogeneic MSCs were demonstrated in the Trident Study, in the treatment of ischemic cardiomyopathy with transendocardial injections [18], in the TOPCARE-DCM pilot trial of patients with BMNC cell suspension for non-ischemic dilated cardiomyopathy [19], and in the Poseidon trial, notwithstanding the small sample size and absence of a placebo group in the latter [20].

Vrtovec’s study utilizing transendocardial injection of CD34^+^ cells for the treatment of ischemic cardiomyopathy achieved an 8% improvement of LVEF (*p* < 0.001) and similar improvements in non-ischemic dilated cardiomyopathy [21]. These are similar to our results and support the treatment of ischemic and non-ischemic dilated cardiomyopathies with autologous CD34^+^ cells.

### What are the probable mechanisms by which ACP-01 improve cardiac performance, and what advantages may ACP-01 offer in the setting of cardiomyopathy?

Following cardiac injury, implantation of ACP-01 improves microcirculation by release of paracrine factors that promote angiogenesis and cell migration, and which minimize the area of ischemic myocardium, thereby rescuing penumbra and lessening dysfunctional macroscopic remodeling of the surrounding non-ischemic myocardium [9, 22]. ACP-01 appear to mitigate pathological remodeling and ischemia of the injured heart [5] in four ways. First, ACP-01 improve microcirculation through angiogenesis. ACP-01 are programmed to form endothelial cells. ACP-01 also have a potent paracrine effect, secreting VEGF, and angiogenin. CXCL8 expression is upregulated by the CXC12 and CXCR4 axis in endothelial cells [23]. Upregulation of the expression of CXCL8 enhances angiogenesis through Ras-MAPK/PI3K activation and the AP-1/NF-kB axis, promoting the proliferation, growth, and viability of vascular endothelial cells [24, 25] (see Central Illustration). Elevated CXCL8 concentrations also mobilize peripheral CD34^+^ precursor cells to amplify the angiogenic response [26]. The second way in which ACP-01 minimize ischemic injury to the myocardium is through cell migration. ACP-01 are strongly attracted to specific cytokines released from injured tissue, resulting in robust migration and embedding of transplanted cells into injured myocardium [5]. For instance, a rodent study demonstrated 29 ACP-01/0.2 mm^2^ sections (i.e., > 4050 cells/mm^3^) in the myocardium [5]. Embedded ACP-01 support tissue survival. The third way in which ACP-01 minimize pathological remodeling of injured myocardium is the action of CXCL8, which attracts immature myeloid and monocytic cells to the area of injury [25], preferentially adopting the “non-inflammatory M2 phenotypical state” to effect modulation and reduction of scarring [27]. Fourth, increased expression of CXCL8 activates CXCR1 and CXCR2 membrane receptors, resulting in downstream translocation of the transcription factors NF-kB to the nucleus and subsequent expression of anti-apoptotic factors [24, 28].

There are other advantages to the ACP-01. Autologous ACP-01 are not subject to cell-to-cell interactions [7, 29], nor immune rejection from alloreactive antibodies [30], and may have longer survival than allogeneic cells [31]. The production and sorting method of autologous ACP-01 results in fewer of the heterogeneous cell populations which can negatively affect therapeutic results [32], and fresh autologous cells may be more effective than stored cells [33].

There is also concern that the homing ability of MSCs may deteriorate with continuous cell division [34]. In that implantation at the site of the target lesion is considered an important determinant of the therapeutic efficacy of MSC therapy, there is evidence of decreased expression of some chemokine receptors (such as CXCR2 and CXCR4) during the continuous passage of MSC, which results in the decreased homing ability of MSCs to target lesions [35].

### What is the safety profile for cardiac stem cell transplantation?

Clinical studies have demonstrated that autologous and allogeneic stem cells are safe, and exhibit few treatment-related adverse events in comparison with control groups. The large, published studies have reported no major adverse events related to cell infusion. In this study, with the exception of the patient with the unrecognized silent MI suffering a technique-related complication, there were no known injuries to target vessels, no heart failure hospitalizations, and no re-infarction. The complications seen are in keeping with other large clinical trials. A study of patients with non-ischemic dilated cardiomyopathy reported heart failure in 5% of the stem cell group as opposed to 18% of the controls (*p* < 0.03) [36]; other studies have reported worsening heart failure with re-hospitalization in approximately 20.0% of patients [15, 18, 37–39].

### Limitations of the study

The implantation of autologous ACP-01 in this study was performed over a decade ago for the treatment of ischemic and non-ischemic dilated cardiomyopathy within a compassionate use protocol. The cell implantations were performed without formal regulatory oversight, and there was no systematic data collection. Data were thus incomplete for many patients. The present IRB approved study is a retrospective outcome analysis of the data derived from the charts of those patients. The assurance that each patient had received maximal medical treatment was required in the consent form; however, there was no supervision by a third party. While the cohort of patients was consecutive, some patients were excluded from the statistical outcomes analysis on the basis of an incomplete or missing certificate of analysis of the ACP-01, because of missing LVEF measurements, or because the patient fell outside the parameters of this study (some patients had pre-implantation LVEF > 50%). The patient population included ischemic and non-ischemic dilated cardiomyopathy and four patients in whom the specific type of cardiomyopathy was uncertain; moreover, the possibility of secondary cardiomyopathies was not examined. The primary and secondary endpoints were limited to LVEF, safety, and patient-reported quality of life assessments. Unfortunately, a standardized, validated quality of life instrument was not used at the time of treatment. Therefore, we were unable to establish reliable assessments of quality of life. Extensive follow-up studies were conducted at different intervals after cell implantation, and some data points were missing. MUGA scans were deemed the most accurate means of measuring LVEF and provided the majority of LVEF determinations. In other cases, the LVEF was determined by echocardiography and in a few by SPECT. Standard tests such as the 6-min walk tests, New York Health Association (NYHA) functional class, and well-validated quality of life analyses were not performed.

## Conclusion

The results of this retrospective study should be viewed with caution given the limitations. However, minimally invasive, transcatheter, intra-myocardial, and intracoronary implantation of autologous ACP-01 is safe and appears to be efficacious in the treatment of ischemic and non-ischemic cardiomyopathy. In the context of the previous clinical trials with ACP-01, there are sufficient grounds to proceed with a prospective, blinded, and randomized phase III clinical trial of ACP-01 implantation, to confirm its efficacy in the treatment for ischemic and non-ischemic cardiomyopathy.

## Data Availability

The data that support the findings of this study are available from Hemostemix but restrictions apply to the availability of these data, which were used under permission for the current study, and so are not publicly available. Data are, however, available from the authors upon reasonable request and with permission of Hemostemix.

## Abbreviations

Ac-LDL: Acetyl low-density lipoprotein
ACP-01: Angiogenic cell precursor
bFGf: Basic fibroblast growth factor
BMNCs: Bone marrow mononuclear cells
CAD: Coroanary artery disease
CCTRN: Cardiovascular Cell Therapy Research Network
CXCL8: Cytokine interleukin-8
ECM: Extracellular matrix
hiPSC-CMs: Human-induced pluripotent stem cell-derived cardiomyocytes
HSCs: Hematopoietic stem cells
LV: Left ventricular
LVEDV: Left ventricular end-diastolic volume
LVEF: Left ventricular ejection fraction
MSCs: Mesenchymal stem cells
MI: Myocardial infarction
MUGA: Multi-gated acquisition scan
NYHA: New York Health Association
PBMCs: Peripheral blood mononuclear cells
proBNP: N-terminal pro-B-type natriuretic peptide
SAEs: Serious adverse events
SCP: Synergetic cell population
SE: Standard error
SPECT: Single-photon emission computerized tomography
VEGF: Vascular endothelial growth factor

## References

[1] Virani SS, Alonso A (2021). American heart association council on epidemiology and prevention statistics committee and stroke statistics subcommittee. Heart disease and stroke statistics-2021 update: a report from the American Heart Association. Circulation.

[2] Conrad N, Judge A, Canoy D, Tran J, Pinho-Gomes AC, Millett ERC, Salimi-Khorshidi G, Cleland JG, McMurray JJV, Rahimi K (2019). Temporal trends and patterns in mortality after incident heart failure: a longitudinal analysis of 86 000 individuals. JAMA Cardiol.

[3] Bittira B, Shum-Tim D, Al-Khaldi A, Chiu RCJ (2003). Mobilization and homing of bone marrow stromal cells in myocardial infarction. Eur J Cardiothorac Surg.

[4] Tse H-F, Kwong Y-L, Chan JKF, Lo G, Ho C-L, Lau C-P (2003). Angiogenesis in ischaemic myocardium by intramyocardial autologous bone marrow mononuclear cell implantation. Lancet.

[5] Porat Y, Porozov S, Belkin D (2006). Isolation of an adult blood-derived progenitor cell population capable of differentiation into angiogenic, myocardial and neural lineages. Br J Haematol.

[6] Balsam LB, Wagers AJ, Christensen JL, Kofidis T, Weissman IL, Robbins RC (2004). Haematopoietic stem cells adopt mature haematopoietic fates in ischaemic myocardium. Nature.

[7] Razeghian-Jahromi I, Matta AG, Canitrot R, Zibaeenezhad MJ, Razmkhah M, Safari A, Nader V, Roncalli J (2021). Surfing the clinical trials of mesenchymal stem cell therapy in ischemic cardiomyopathy. Stem Cell Res Ther.

[8] Al-Khalaf HH, Al-Harbi B, Al-Sayed A, Arafah M, Tulbah A, Jarman A, Al-Mohanna F, Aboussekhra A (2019). Interleukin-8 activates breast cancer-associated adipocytes and promotes their angiogenesis- and tumorigenesis-promoting effects. Mol Cell Biol.

[9] Sun Z, Wu J, Fujii H (2008). Human angiogenic cell precursors restore function in the infarcted rat heart: a comparison of cell delivery routes. Eur J Heart Fail.

[10] Arom KV, Ruengsakulrach P, Jotisakulratana V (2008). Intramyocardial angiogenic cell precursor injection for cardiomyopathy. Asian Cardiovasc Thorac Ann.

[11] Williams AR, Trachtenberg B, Velazquez D (2011). Intramyocardial stem cell injection in patients with ischemic cardiomyopathy functional recovery and reverse remodeling. Circ Res.

[12] Chaithiraphan S, Dutsadeevettakul S, Tasneeyapant S (2009). Transcoronary injection of angiogenic cells precursors in autologous stem CELL IN ischemic cardiomyopathy: a clinical study of 106 cases in Thailand. Asean Heart Journal.

[13] Traverse JH, Henry TD, Ellis SG (2011). Effect of intracoronary delivery of autologous bone marrow mononuclear cells 2 to 3 weeks following acute myocardial infarction on left ventricular function: the LateTIME randomized trial. JAMA.

[14] Perin EC, Willerson JT, Pepine CJ (2012). Effect of transendocardial delivery of autologous bone marrow mononuclear cells on functional capacity, left ventricular function, and perfusion in chronic heart failure: the FOCUS-CCTRN trial. JAMA.

[15] Choudry F, Hamshere S, Saunders N (2016). A randomized double-blind control study of early intra-coronary autologous bone marrow cell infusion in acute myocardial infarction: the REGENERATE-AMI clinical trial. Eur Heart J.

[16] Martino H, Brofman P, Greco O (2015). Multicentre, randomized, double-blind trial of intracoronary autologous mononuclear bone marrow cell injection in non-ischaemic dilated cardiomyopathy (the dilated cardiomyopathy arm of the MiHeart study). Eur Heart J.

[17] Henry TD, Pepine CJ, Lambert CR (2017). The Athena trials: Autologous adipose-derived regenerative cells for refractory chronic myocardial ischemia with left ventricular dysfunction. Catheter Cardiovasc Interv.

[18] Florea V, Rieger AC, DiFede DL (2017). Dose comparison study of allogeneic mesenchymal stem cells in patients with ischemic cardiomyopathy (The TRIDENT Study). Circ Res.

[19] Leistner DM, Fischer-Rasokat U, Honold J (2011). Transplantation of progenitor cells and regeneration enhancement in acute myocardial infarction (TOPCARE-AMI): final 5-year results suggest long-term safety and efficacy. Clin Res Cardiol.

[20] Bolli R, Solankhi M, Tang XL, Kahlon A (2022). Cell therapy in patients with heart failure: a comprehensive review and emerging concepts. Cardiovasc Res.

[21] Vrtovec B, Poglajen G, Lezaic L (2013). Comparison of transendocardial and intracoronary CD34+ cell transplantation in patients with nonischemic dilated cardiomyopathy. Circulation.

[22] Banovic M, Poglajen G, Vrtovec B, Ristic A (2022). Contemporary challenges of regenerative therapy in patients with ischemic and non-ischemic heart failure. J Cardiovasc Dev Dis.

[23] Perbellini O, Cioffi F, Malpeli G, Zanolin E, Lovato O, Scarpa A (2015). Up-regulation of CXCL8/interleukin-8 production in response to CXCL12 in chronic lymphocytic leukemia. Leuk Lymphoma.

[24] Karin M, Lin A (2002). NF-κB at the crossroads of life and death. Nat Immunol.

[25] Han Z-J, Li Y-B, Yang L-X, Cheng H-J, Liu X, Chen H (2021). Roles of the CXCL8-CXCR1/2 axis in the tumor microenvironment and Immunotherapy. Molecules.

[26] Massa M, Rosti V, Ferrario M (2005). Increased circulating hematopoietic and endothelial progenitor cells in the early phase of acute myocardial infarction. Blood.

[27] Schömig K, Busch G, Steppich B (2006). Interleukin-8 is associated with circulating CD133+ progenitor cells in acute myocardial infarction. Eur Heart J.

[28] Jha NK, Jha SK, Kar R, Nand P, Swati K, Goswami VK (2019). Nuclear factor-kappa B as a therapeutic target for Alzheimer’s disease. J Neurochem.

[29] Brodarac A, Šarić T, Oberwallner B (2015). Susceptibility of murine induced pluripotent stem cell-derived cardiomyocytes to hypoxia and nutrient deprivation. Stem Cell Res Ther.

[30] Hare JM, Fishman JE, Gerstenblith G (2012). Comparison of allogeneic vs autologous bone marrow-derived mesenchymal stem cells delivered by transendocardial injection in patients with ischemic cardiomyopathy: the POSEIDON randomized trial. JAMA.

[31] Burst VR, Gillis M, Pütsch F (2009). Poor cell survival limits the beneficial impact of mesenchymal stem cell transplantation on acute kidney injury. Nephron Exp Nephrol.

[32] Ikebe C, Suzuki K (2014). Mesenchymal stem cells for regenerative therapy: optimization of cell preparation protocols. Biomed Res Int.

[33] Mathiasen AB, Jørgensen E, Qayyum AA, Haack-Sørensen M, Ekblond A, Kastrup J (2012). Rationale and design of the first randomized, double-blind, placebo-controlled trial of intramyocardial injection of autologous bone-marrow derived Mesenchymal Stromal Cells in chronic ischemic Heart Failure (MSC-HF Trial). Am Heart J.

[34] Rombouts WJ, Ploemacher RE (2003). Primary murine MSC show highly efficient homing to the bone marrow but lose homing ability following culture. Leukemia.

[35] Chen H, Zhou L (2022). Treatment of ischemic stroke with modified mesenchymal stem cells. Int J Med Sci.

[36] Vrtovec B, Poglajen G, Lezaic L (2013). Effects of intracoronary CD34+ stem cell transplantation in nonischemic dilated cardiomyopathy patients: 5-year follow-up. Circ Res.

[37] Jackson KA, Majka SM, Wang H (2001). Regeneration of ischemic cardiac muscle and vascular endothelium by adult stem cells. J Clin Invest.

[38] Perin EC, Dohmann HFR, Borojevic R (2003). Transendocardial, autologous bone marrow cell transplantation for severe, chronic ischemic heart failure. Circulation.

[39] Alvarez PA, Schwarz ER, Ramineni R (2013). Periprocedural adverse events in cell therapy trials in myocardial infarction and cardiomyopathy: a systematic review. Clin Res Cardiol.

